# Complete genome sequence of *Gordonia bronchialis* type strain (3410^T^)

**DOI:** 10.4056/sigs.611106

**Published:** 2010-01-28

**Authors:** Natalia Ivanova, Johannes Sikorski, Marlen Jando, Alla Lapidus, Matt Nolan, Susan Lucas, Tijana Glavina Del Rio, Hope Tice, Alex Copeland, Jan-Fang Cheng, Feng Chen, David Bruce, Lynne Goodwin, Sam Pitluck, Konstantinos Mavromatis, Galina Ovchinnikova, Amrita Pati, Amy Chen, Krishna Palaniappan, Miriam Land, Loren Hauser, Yun-Juan Chang, Cynthia D. Jeffries, Patrick Chain, Elizabeth Saunders, Cliff Han, John C. Detter, Thomas Brettin, Manfred Rohde, Markus Göker, Jim Bristow, Jonathan A. Eisen, Victor Markowitz, Philip Hugenholtz, Hans-Peter Klenk, Nikos C. Kyrpides

**Affiliations:** 1 DOE Joint Genome Institute, Walnut Creek, California, USA; 2 DSMZ - German Collection of Microorganisms and Cell Cultures GmbH, Braunschweig, Germany; 3 Los Alamos National Laboratory, Bioscience Division, Los Alamos, New Mexico, USA; 4 Biological Data Management and Technology Center, Lawrence Berkeley National Laboratory, Berkeley, California, USA; 5 Oak Ridge National Laboratory, Oak Ridge, Tennessee, USA; 6 HZI – Helmholtz Centre for Infection Research, Braunschweig, Germany; 7 University of California Davis Genome Center, Davis, California, USA

**Keywords:** Obligate aerobic, human-pathogenic, endocarditis, Gram-positive, non-motile, *Gordoniaceae*

## Abstract

*Gordonia bronchialis* Tsukamura 1971 is the type species of the genus. *G. bronchialis* is a human-pathogenic organism that has been isolated from a large variety of human tissues. Here we describe the features of this organism, together with the complete genome sequence and annotation. This is the first completed genome sequence of the family *Gordoniaceae*. The 5,290,012 bp long genome with its 4,944 protein-coding and 55 RNA genes is part of the *** G****enomic* *** E****ncyclopedia of* *** B****acteria and* *** A****rchaea * project.

## Introduction

Strain 3410^T^ (= DSM 43247 = ATCC 25592 = JCM 3198) is the type strain of the species *Gordonia bronchialis*, which is the type species of the genus. The genus *Gordonia* (formerly *Gordona*) was originally proposed by Tsukamura in 1971 [1]. The generic name *Gordona* has been chosen to honor Ruth E. Gordon, who studied extensively ‘*Mycobacterium*’ *rhodochrous* (included later as a member of *Gordona*) [1]. In 1977, it was subsumed into the genus *Rhodococcus* [2], but revived again in 1988 by Stackebrandt *et al*. [3]. At the time of writing, the genus contained 28 validly published species [4]. The genus *Gordonia* is of great interest for its bioremediation potential [5]. Some species of the genus have been used for the decontamination of polluted soils and water [6,7]. Other species were isolated from industrial waste water [8], activated sludge foam [9], automobile tire [10], mangrove rhizosphere [11], tar-contaminated oil [12], soil [13] and an oil-producing well [7]. Further industrial interest in *Gordonia* species stems from their use as a source of novel enzymes [14,15]. There are, however, quite a number of *Gordonia* species that are associated with human and animal diseases [16], among them *G. bronchialis*. Here we present a summary classification and a set of features for *G. bronchalis* 3410^T^, together with the description of the complete genomic sequencing and annotation.

## Classification and features

Strain 3410^T^ was isolated from the sputum of a patient with pulmonary disease (probably in Japan) [1]. Further clinical strains in Japan have been isolated from pleural fluid, tumor in the eyelid, granuloma, leukorrhea, skin tissue and pus [17]. In other cases, *G. bronchialis* caused bacteremia in a patient with a sequestrated lung [18] and a recurrent breast abscess in an immunocompetent patient [19]. Finally, *G. bronchialis* was isolated from sternal wound infections after coronary artery bypass surgery [20]. *G. bronchialis* shares 95.8-98.7% 16S rRNA gene sequence similarity with the other type strains of the genus *Gordonia*, and 95.3-96.4% with the type strains of the neighboring genus *Williamsia*.

**Figure 1:**
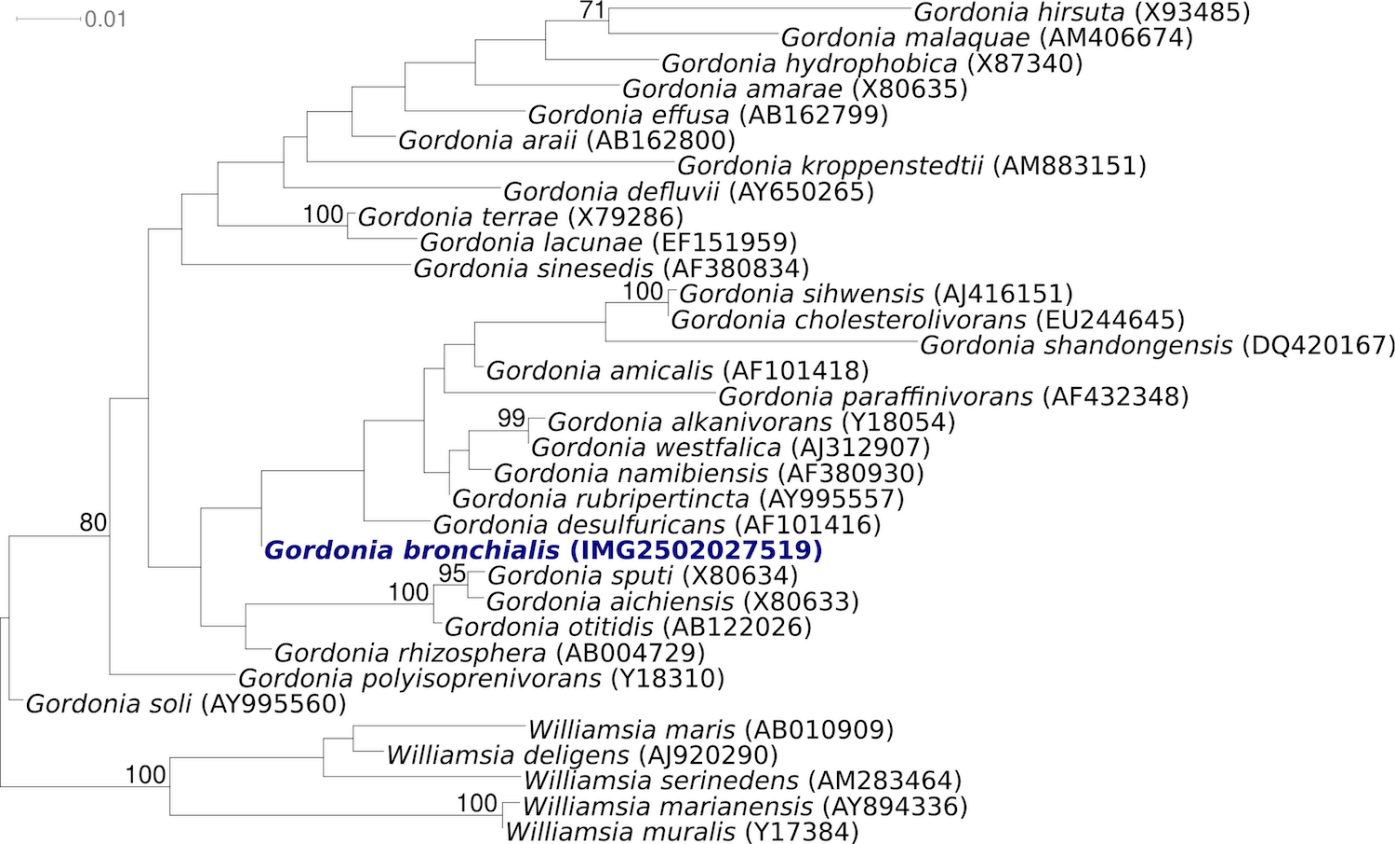
Phylogenetic tree highlighting the position of *G. bronchialis* 3410^T^ relative to the other type strains within the genus *Gordonia*. The tree was inferred from 1,446 aligned characters [21,22] of the 16S rRNA gene sequence under the maximum likelihood criterion [23] and rooted with the type strains of the neighboring genus *Williamsia*. The branches are scaled in terms of the expected number of substitutions per site. Numbers above branches are support values from 1,000 bootstrap replicates if larger than 60%. Lineages with type strain genome sequencing projects registered in GOLD [24] are shown in blue, published genomes in bold.

Figure 1 shows the phylogenetic neighborhood of for *G. bronchialis* 3410^T^ in a 16S rRNA based tree. The sequences of the two 16S rRNA gene copies in the genome of *G. bronchialis* 3410^T^, differ from each other by one nucleotide, and differ by up to 5 nucleotides from the previously published 16S rRNA sequence from DSM 43247 (X79287). These discrepancies are most likely due to sequencing errors in the latter sequence.

In a very comprehensive study, Tsukamura analyzed a set of 100 quite diverse characters for 41 *G. bronchialis* strains isolated from sputum of patients with pulmonary disease, including the type strain [1]. Unfortunately, this study does not present the characteristics of the type strain 3410^T^ as such. We nevertheless first present these data, as this study gives a good overview of the species itself. In order to summarize the data here, we regard positive reactions in more than 34 strains as positive, and positive reactions in only 13 or less strains as negative. Most characters, however, are either clearly positive (40 or 41 strains) or clearly negative (0 or 1 strains). The detailed methods are reported elsewhere [25,26].

**Figure 2:**
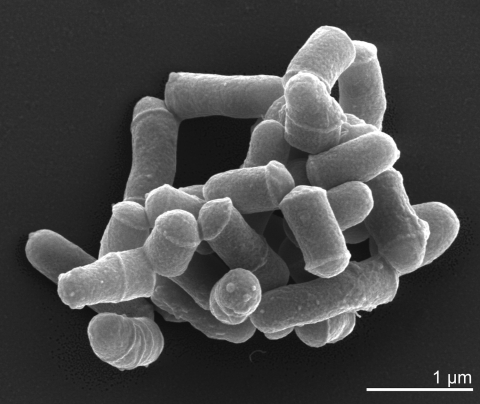
Scanning electron micrograph of *G.  bronchialis* 3410^T^

*G. bronchialis* is Gram-positive (Table 1) and shows slight but not strong acid-fastness. A mycelium is not observed. *G. bronchialis* strains are non-motile and produce neither conidia nor endospores [1,3]. *G. bronchialis* is an obligately aerobic chemoorganotroph with an oxidative-type metabolism [3].The cells are rod-shaped and show compact grouping (like a cord) (Figure 2), and provide a rough colonial morphology with pinkish-brown colony pigmentation [1]. Photochromogenicity was not observed. *G. bronchialis* grows quite rapidly [1], with visible colonies appearing within 1-3 days [1,36]. *G. bronchialis* is positive for catalase and nitrate reduction, but arylsulphatase (3 days and 2 weeks), salicylate and PAS degradation was not observed [1]. Growth occurs on 0.2% sodium p-aminosalicylate and 62.5 and 125 µg NH_2_OH-HCl/ml, but not with 250 or 500 µg. *G. bronchialis* is tolerant to both 0.1 and 0.2% picric acid. *G. bronchialis* grows at 28°C and 37°C, but not at 45°C or 52°C [1]. *G. bronchialis* is positive for acetamidase, urease, nicotinamidase and pyrazinamidase, but negative for benzamidase, isonicotinamidase, salicylamidase, allanoinase, succinamidase, and malonamidase [1]. *G. bronchialis* utilizes acetate, succinate, malate, pyruvate, fumarate, glycerol, glucose, mannose, trehalose, inositul, fructose, sucrose, ethanol, propanol, and propylene glycol as a carbon source for growth, but not citrate, benzoate, malonate, galactose, arabinose, xylose, rhamnose, raffinose, mannitol, sorbitol, or various forms of butylene glycol (1,3-; 1,4-; 2,3-) [1]. *G. bronchialis* utilizes L-glutamate and acetamide as a N-C source, but not L-serine, benzamide, monoethanolamine or trimethylene diamine. Glucosamine is utilized by 18 strains [1]. *G. bronchialis* utilizes as nitrogen source L-glutamate, L-serine, L-methionine, acetamide, urea, pyrazinamide, isonicotinamide, nicotinamide, succinamide, but not benzamide and nitrite. Nitrate is utilized by 25 strains as nitrogen source [1]. *G. bronchialis* strains do not produce nicotinic acid. *G. bronchialis* strains do not grow on TCH medium (10 µg/ml) or on salicylate medium (0.05% and 0.01%) [1].

**Table 1:** Classification and general features of *G. bronchialis* 3410^T^ according to the MIGS recommendations [27]

MIGS ID	Property	Term	Evidence code
	Current classification	Domain *Bacteria*	TAS [28]
		Phylum *Actinobacteria*	TAS [29]
		Class *Actinobacteria*	TAS [30]
		Order *Actinomycetales*	TAS [30]
		Suborder *Corynebacterineae*	TAS [30,31]
		Family *Gordoniaceae*	TAS [30]
		Genus *Gordonia*	TAS [3,30,32]
		Species *Gordonia bronchialis*	TAS [1]
		Type strain 3410	TAS [1]
	Gram stain	positive	TAS [1]
	Cell shape	short rods in compact grouping (cord-like)	TAS [1]
	Motility	non-motile	TAS [1]
	Sporulation	non-sporulating	TAS [1]
	Temperature range	grows at 28°C and 37°C, not at 45°C	TAS [1]
	Optimum temperature	probably between 28°C and 37°C	TAS [1]
	Salinity	2.5%	TAS [33]
MIGS-22	Oxygen requirement	obligate aerobe	TAS [1]
	Carbon source	mono- and disaccharides	TAS [1]
	Energy source	chemoorganotroph	TAS [3]
MIGS-6	Habitat	human	TAS [1]
MIGS-15	Biotic relationship	free living	NAS
MIGS-14	Pathogenicity	opportunistic pathogen	TAS [1,17-20]
	Biosafety level	2	TAS [34]
	Isolation	sputum from human with pulmonary disease in (probably) Japan	TAS [1]
MIGS-4	Geographic location	global	TAS [1,17-20]
MIGS-5	Sample collection time	1971 or before	TAS [1]
MIGS-4.1 MIGS-4.2	Latitude, Longitude	not reported	
MIGS-4.3	Depth	not reported	
MIGS-4.4	Altitude	not reported	

Evidence codes - IDA: Inferred from Direct Assay (first time in publication); TAS: Traceable Author Statement (i.e., a direct report exists in the literature); NAS: Non-traceable Author Statement (i.e., not directly observed for the living, isolated sample, but based on a generally accepted property for the species, or anecdotal evidence). These evidence codes are from of the Gene Ontology project [35]. If the evidence code is IDA, then the property was directly observed for a live isolate by one of the authors or an expert mentioned in the acknowledgements.

In the following, characteristics of the type strain 3410^T^ are presented: strain 3410^T^ reduces nitrate and hydrolyses urea, but it does not hydrolyze aesculin, allantoin or arbutin [37]. It decomposes (%, w/v) starch (1) and uric acid (0.5), but not hypoxanthine (0.4), tributyrin (0.1), tween 80 (1), tyrosine (0.5) and xanthine (0.4) [37]. It grows on glycerol (1) and sodium fumarate (1) as sole carbon sources (%, w/v), but not on arbutin (1), D-cellobiose (1), N-acetyl-D-glucosamine (0.1), adipic acid (0.1), betaine (0.1), oxalic acid (0.1), propan-1-ol (0.1) [37]. Strain 3410^T^ grows in the presence (% w/v) of oleic acid (0.1) and zinc chloride (0.001) [37].

In an API ZYM test, strain 3410^T^ reacts positively for alkaline phosphatase, butyrate esterase, leucine arylamidase and naphtol-AS-BI-phosphohydrolase, but not for caprylate esterase, cystine arylamidase, β-glucosidase, myristate lipase, and valine arylamidase [13]. Complementary to the results of Tsukamura [1], strain 3410^T^ utilizes as sole carbon source **D**(+) cellobiose, **D**(+) galactose, **D**(+) mannose, meso-inositol, **L**(+) rhamnose and sodium succinate, but not **D**(-)lactose, **D**(-) ribose, sodium benzoate and sodium citrate [38]. The use of **D**(+) galactose [38] might contrast above reported results from Tsukamura [1]. **L**-threonine and **L**-valinine are used as sole nitrogen source by strain 3410^T^, but not **L**-asparagine, **L**-proline and **L**-serine [38]. Interestingly, Tsukamura reports that 40 out of 41 strains utilize L-serine as sole nitrogen source [1], and it is not clear if the only negative strain in the Tsukamura study could be the type strain 3410^T^ [1].

In the BiOLOG system, strain 3410^T^ reacts positively for α-cyclodextrin, β-cyclodextrin, dextrin, glycogen, maltose, maltotriose, **D** -mannose, 3-methyl glucose, palatinose, **L**-raffinose, salicin, turanose, **D**-xylose, **L**-lactic acid, methyl succinate, *N*-acetyl- **L**-glutamic acid [12], but not for *N*-acetylglucosamine, amygdalin, **D**-arabitol, **L**-Rhamnose, **D**-ribose, **D**-sorbitol, **D**-trehalose, acetic acid, α-hydroxybutyric acid, β-hydroxybutyric acid, α-ketoglutaric acid, α-ketovaleric acid, **L**-lactic acid methyester, **L**-malic acid, propionic acid, succinamic acid, alaninamide, **L**-alanine and glycerol [12]. Further carbon source utilization results are published elsewhere [8].

Drug susceptibility profiles of 13 *G. bronchialis* strains from clinical samples have been examined in detail [17], but they are too complex to summarize here. No significant matches with any 16S rRNA sequences from environmental genomic samples and surveys are reported at the NCBI BLAST server (November 2009).

### Chemotaxonomy

The cell-wall peptidoglycan is based upon meso-diaminopimelic acid (variation Alγ). The glycan moiety of the peptidoglycan contains *N*-glycolylmuramic acid. The wall sugars are arabinose and galactose. Mycolic acids are present with a range of ca. 48-66 carbon atoms. The predominant menaquinone is MK-9(H2), with only low amounts of MK-9(H0), MK-8(H2), and MK-7(H2) [3,8,39-41]. Moreover, the cell envelope of *G. bronchialis* 3410^T^ contains a lipoarabinomannan-like lipoglycan [42]. The same study also observed a second amphiphilic fraction with properties suggesting a phosphatidylinositol mannoside [42]. The cellular fatty acid composition (%) is C_16:0_ (23), tuberculostearic acid (20), C_16:1cis9_ (16), C_16:1cis7_ (11), C_18:1_ (10), and 10-methyl C_17:0_ (7). All other fatty acids are at 3% or below [8].

## Genome sequencing and annotation

### Genome project history

This organism was selected for sequencing on the basis of its phylogenetic position, and is part of the *** G****enomic* *** E****ncyclopedia of* *** B****acteria and* *** A****rchaea * project. The genome project is deposited in the Genome OnLine Database [24] and the complete genome sequence is deposited in GenBank. Sequencing, finishing and annotation were performed by the DOE Joint Genome Institute (JGI). A summary of the project information is shown in Table 2.

**Table 2:** Genome sequencing project information

**MIGS ID**	**Property**	**Term**
MIGS-31	Finishing quality	Finished
MIGS-28	Libraries used	Two Sanger libraries: 8kb pMCL200 and fosmid pcc1Fos One 454 Pyrosequence standard library
MIGS-29	Sequencing platforms	ABI3730, 454 GS FLX
MIGS-31.2	Sequencing coverage	7.98× Sanger; 23.2× Pyrosequence
MIGS-30	Assemblers	Newbler, phrap
MIGS-32	Gene calling method	Prodigal, GenePRIMP
	INSDC ID	CP001802
	GenBank Date of Release	October 28, 2009
	GOLD ID	Gc01134
	NCBI project ID	29549
	Database: IMG-GEBA	2501939625
MIGS-13	Source material identifier	DSM 43247
	Project relevance	Tree of Life, GEBA

### Growth conditions and DNA isolation

*G. bronchialis* 3410^T^, DSM 43247, was grown in DSMZ 535 [43] at 28°C. DNA was isolated from 1-1.5 g of cell paste using Qiagen Genomic 500 DNA Kit (Qiagen, Hilden, Germany) following the manufacturer's instructions with modification st/LALMP for cell lysis according to Wu *et al*. [44].

### Genome sequencing and assembly

The genome was sequenced using a combination of Sanger and 454 sequencing platforms. All general aspects of library construction and sequencing performed at the JGI can be found on the JGI website. 454 Pyrosequencing reads were assembled using the Newbler assembler version 1.1.02.15 (Roche). Large Newbler contigs were broken into 5,776 overlapping fragments of 1,000 bp and entered into assembly as pseudo-reads. The sequences were assigned quality scores based on Newbler consensus q-scores with modifications to account for overlap redundancy and to adjust inflated q-scores. A hybrid 454/Sanger assembly was made using the parallel phrap assembler (High Performance Software, LLC). Possible mis-assemblies were corrected with Dupfinisher [45] or transposon bombing of bridging clones (Epicentre Biotechnologies, Madison, WI). Gaps between contigs were closed by editing in Consed, custom primer walk or PCR amplification. A total of 876 primer walk reactions, 12 transposon bombs, and 1 pcr shatter libraries were necessary to close gaps, to resolve repetitive regions, and to raise the quality of the finished sequence. The error rate of the completed genome sequence is less than 1 in 100,000. Together all sequence types provided 51.2 × coverage of the genome. The final assembly contains 52,329 Sanger and 508,130 pyrosequence reads.

### Genome annotation

Genes were identified using Prodigal [46] as part of the Oak Ridge National Laboratory genome annotation pipeline, followed by a round of manual curation using the JGI GenePRIMP pipeline [47]. The predicted CDSs were translated and used to search the National Center for Biotechnology Information (NCBI) nonredundant database, UniProt, TIGRFam, Pfam, PRIAM, KEGG, COG, and InterPro databases. Additional gene prediction analysis and manual functional annotation was performed within the Integrated Microbial Genomes Expert Review (IMG-ER) platform [48].

## Genome properties

**Figure 3:**
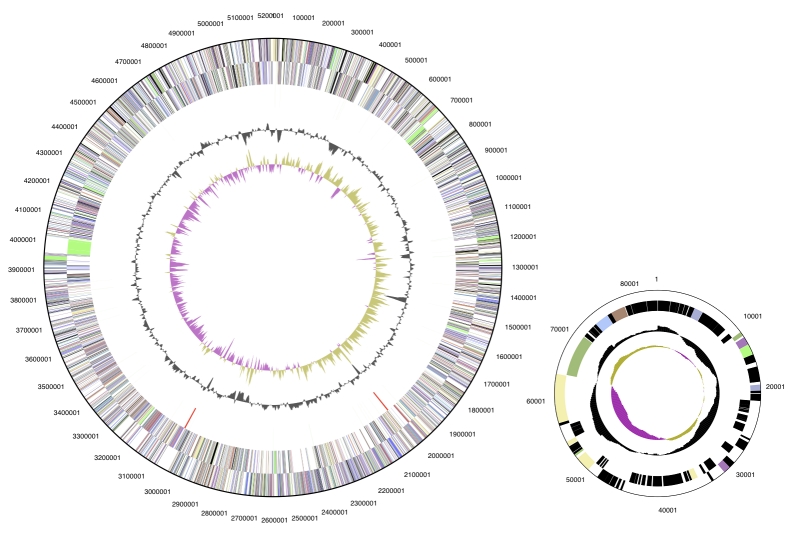
Graphical circular map of the chromosome and the plasmid. From outside to the center: Genes on forward strand (color by COG categories), Genes on reverse strand (color by COG categories), RNA genes (tRNAs green, rRNAs red, other RNAs black), GC content, GC skew.

The genome consists of a 5.2 Mbp long chromosome and a 81,410 bp plasmid (Table 3 and Figure 3). Of the 4,999 genes predicted, 4,944 were protein coding genes, and 55 RNAs; 264 pseudogenes were also identified. The majority of the protein-coding genes (69.1%) were assigned with a putative function while those remaining were annotated as hypothetical proteins. The distribution of genes into COGs functional categories is presented in Table 4.

**Table 3:** Genome Statistics

**Attribute**	**Value**	**% of Total**
Genome size (bp)	5,290,012	100.00%
DNA coding region (bp)	4,897,508	92.58%
DNA G+C content (bp)	3,546,559	67.04%
Number of replicons	1	
Extrachromosomal elements	1	
Total genes	4,999	100.00%
RNA genes	55	1.10%
rRNA operons	2	
Protein-coding genes	4,944	98.90%
Pseudo genes	264	5.28%
Genes with function prediction	3,453	69,07%
Genes in paralog clusters	804	16.08%
Genes assigned to COGs	3,335	66.71%
Genes assigned Pfam domains	3,508	70.17%
Genes with signal peptides	1,038	20.76%
Genes with transmembrane helices	1,209	24.18%
CRISPR repeats	0	

**Table 4:** Number of genes associated with the general COG functional categories

**Code**	**value**	**%age**	**Description**
J	164	3.3	Translation, ribosomal structure and biogenesis
A	1	0.0	RNA processing and modification
K	357	7.2	Transcription
L	238	4.8	Replication, recombination and repair
B	1	0.0	Chromatin structure and dynamics
D	28	0.6	Cell cycle control, mitosis and meiosis
Y	0	0.0	Nuclear structure
V	50	1.0	Defense mechanisms
T	158	3.2	Signal transduction mechanisms
M	133	2.7	Cell wall/membrane biogenesis
N	2	0.0	Cell motility
Z	1	0.0	Cytoskeleton
W	0	0.0	Extracellular structures
U	30	0.6	Intracellular trafficking and secretion
O	123	2.5	Posttranslational modification, protein turnover, chaperones
C	261	5.3	Energy production and conversion
G	197	4.0	Carbohydrate transport and metabolism
E	283	5.7	Amino acid transport and metabolism
F	90	1.8	Nucleotide transport and metabolism
H	172	3.5	Coenzyme transport and metabolism
I	270	5.5	Lipid transport and metabolism
P	202	4.1	Inorganic ion transport and metabolism
Q	210	4.2	Secondary metabolites biosynthesis, transport and catabolism
R	505	10.2	General function prediction only
S	286	5.8	Function unknown
-	1664	33.7	Not in COGs

## Notes

While the manuscript was in editorial processing a 29^th^ species in the genus *Gordonia* was published: *G. hankookensis* [49], which is not featured in Figure 1.
